# Reflective Journals of Students Taking a Positive Youth-Development Course in a University Context in Hong Kong

**DOI:** 10.1100/2012/131560

**Published:** 2012-08-22

**Authors:** Daniel T. L. Shek, Florence K. Y. Wu

**Affiliations:** ^1^Department of Applied Social Sciences, The Hong Kong Polytechnic University, Hong Kong; ^2^Public Policy Research Institute, The Hong Kong Polytechnic University, Hong Kong; ^3^Department of Social Work, East China Normal University, Shanghai 200241, China; ^4^Kiang Wu Nursing College of Macau, Macau; ^5^Division of Adolescent Medicine, Department of Pediatrics, Kentucky Children's Hospital, University of Kentucky College of Medicine, Lexington, KY 40506-9983, USA; ^6^Department of Educational Psychology, The Chinese University of Hong Kong, Hong Kong

## Abstract

To promote the holistic development of university students, a course entitled “Tomorrow's Leaders” was developed and offered at The Hong Kong Polytechnic University. Based on a case study approach, reflective journals of five outstanding students of the course are presented and analyzed (i.e., thick description), with several themes emerging from the reflection. First, the students liked the course, and they identified many positive attributes. Second, the students appreciated the instructors. Third, the students viewed that the course contributed to different aspects of their development. Fourth, some areas of improvements were proposed. In conjunction with other evaluation mechanisms, the present findings strongly suggest that the course is able to promote psychosocial competencies in university students taking this course.

## 1. Introduction

The education system in Hong Kong is undergoing a huge reform. Beginning from 2012/13 academic year, university education in Hong Kong will be changed from a three-year curriculum to a four-year curriculum. How should we nurture university students under the new curriculum? While universities in Hong Kong, normally claim to promote “holistic development” in university students, this is rarely reflected in the credit-bearing courses, where intellectual abilities alone are usually emphasized [1]. Against this background, a course entitled “Tomorrow's Leaders” was developed at The Hong Kong Polytechnic University [2]. The course was piloted in 2010/11 school year, and different evaluation mechanisms were used to evaluate the course, including objective outcome evaluation, subjective outcome evaluation, process evaluation, and qualitative evaluation, based on reflective notes of the students.

 With the generous support and donation of the Wofoo Foundation, scholarships were awarded to five outstanding students in this course. It is argued that by focusing on these “extreme” cases, we can get an additional perspective on the implementation and outcomes of the course. Therefore, these five students were invited to write a personal reflection about the course. After obtaining the consent of the students to disclose their names and with minor language editing, sharing of the five students is included in this paper. Consistent with the spirit of “thick description” in qualitative research, sharing by these five students is included in this paper. The qualitative data were analyzed based on the case study approach. Yin [3] pointed out that a case study “investigates a contemporary phenomenon within its real-life context, especially when the boundaries between phenomenon and context are not clearly evident” (page 13). There are also findings showing that the case study approach is a flexible strategy to understand the reality using different types of data [4, 5].

**Reflective Journal 1 (CHOW Tsz Ho, George)**

The first time I heard of the subject “Tomorrow's Leaders” was probably at the end of August, 2011 on the Common Orientation Day for freshmen. It is now almost one year. The reason why I chose this as my general education subject is not that I want to be a leader but that I want to understand more about leadership.

Leaders are important persons who lead a group of people to finish a job. The group of people can range from a few team mates to several billion people. Leaders are decision makers whose decisions are affecting the development of a team or even a country. It is interesting to know more about the successful traits of these people. Therefore, I enrolled in the subject.

In fact, the subject met my expectation. In the first lecture, the teachers told us that this course was not designed to transform us to brilliant leaders. They were there to provide materials and guide us to have reflection. In the very same lecture, there were many class activities which made the class very interactive and the activities could be articulated into the theories. For instance, there were self-assessments which allowed us to reflect on ourselves in different aspects so that we understood ourselves more and knew how to perform better. The helpful lecturer usually came over to each group to see if we could follow properly.

Incorporated with many meaningful class activities, the lectures were very well organised and the learning materials were very well prepared. There was a lecture outline for each lecture, providing an overview of the lecture. The outlines are good materials for preview and review. The lecture content was on Power Point slides which were also well structured and informative. It included concise explanation for various aspects and vivid examples for the explanation. Through attending the lectures and reading the lecture notes, our understanding towards each leadership competence was deepened.

In order to complete the course, we had to prepare a Group Project Presentation and write an Individual Term Paper. Both assignments provided valuable learning experiences to us. For the Group Project Presentation, we tried our best to introduce one of the leadership competencies—emotional competence. This job was not easy because we had to plan what content to include, what format to use and how much time for each section. After deciding the rough frame of the presentation, we divided it into several parts and made everyone have a role in the construction of the project. I was responsible to think of a way to strengthen the chosen attribute and then told my fellow classmates. During the process, it gave me a feeling of being a teacher. The fact is that preparing a class activity which can articulate your theory is not that easy. There are books written specifically for class activities but they were found to be not suitable because of difficulty in implementation. At that time, I suddenly realized how mighty our lecturer was. On the day of presentation, our group wore full suits to present. In my opinion, we looked very smart and united. During the presentation, we used many different formats to facilitate classmates' understanding and promoted their level of participation. For example, we had dramas, hypothetical situations and questions. We made the presentation both informative and interactive. Every member did a good job.

The Individual Term Paper was a great challenge to all of us. It required us to critically discuss the concept of leadership quality covered in the group presentation on the conceptual level and evaluate the extent to which we possess this leadership quality. We think that was difficult because we are science students. It is unusual for us to discuss conceptual stuff, not to mention to discuss the concept critically. The last resort was, of course, to go to the library to borrow a stack of books related to the topic. Extracting useful information from that stack of books is not an easy job. In the process, I had to look for various definitions of emotional intelligence and emotional competence, look for their virtues and drawbacks, and evaluate myself and cite references. In fact, it was very time-consuming and exhausting, especially at the time of having many deadlines and the final examination was approaching. However, writing the Individual Term Paper gave me a valuable opportunity to have a very deep understanding towards the specific competence chosen. Happily, I got distinction out of my work. This gives me great encouragement and fulfilment.

After finishing the course, I find that it provided a good introduction to various leadership traits and allowed us to reflect on ourselves. I think this is paramount for our personal development. There is a Chinese proverb: Seeing a virtuous person makes you think of being as good as him. How true? If we do not know what the successful traits of good leaders are, how can we be one of them in the future? Therefore, it is good that we have this course in university as a General Education subject.

From my point of view, a good leader must possess his/her own belief and work it out unflinchingly. His belief and his action work together make him a leader with integrity. Only in that way, he can get trust from his supporters who are willing to follow him. Being a good leader is not merely by possessing those leadership traits: self-understanding, emotional competence, cognitive competence, resilience, social competence, positive personal identity, assertiveness, and so forth. Leaders must be passionate, honest, and devoted. Without these qualities, leaders are no different from manipulators.

In conclusion, I would like to thank every teacher who has devoted his/her effort in developing the course. They have made this course very enjoyable and enriching. Moreover, I would also like to thank the donor of Wofoo Foundation Scholarship. The generosity of the Foundation has made the learning atmosphere very active and I am very honoured that the scholarship was granted to me. In the future, I hope more and more junior schoolmates will enrol in this course and learn to be tomorrow's leaders.

**Reflective Journal 2 (Leung Wai Chun)**

As a recipient of Wofoo Foundation Scholarship, it is my pleasure to have this opportunity to write a few words expressing how the subject inspired me and helped my personal development. Furthermore, I would like to share some personal feelings after finishing the subject as well.

The subject facilitates me to be more confident in taking up a leader's role in my social work profession. The course introduced thirteen elements that a successful leader should possess. I have heard some of them before but some of them were new to me. One new element has inspired me a lot and provided me insight to be a leader is “spirituality.” A spiritual leadership style should include spiritual values such as integrity, honesty, and humility. By showing us a movie called “Invictus,” the teacher used Nelson Mandela as an example to demonstrate these elements. During the movie, I was impressed by the decision made by Mandela. He provided meaning (i.e., unity of one nation) for every political decision even though such decision would be at the expense of his political capital. He could create ethical influence and climate that would influence the others. His belief of unity and caring for his original enemies guided him to be the President. This element inspired me that as a social worker, despite facing difficulties and structural constraints, there are some human values that I have to uphold—that is honest communication with self and others and at the same time be respectful and caring to every human, even the one you hate or originally is your enemy. Of course, this type of leadership quality sounds hard to achieve. It is difficult to love the one you hate. However, such leadership practice is attractive to me and I think I am willing to learn to be a spiritual leader in order to make ethical influence to my followers.

Another element that inspired me is resilience. Resilience as a capacity refers to the capacity of an individual for adapting to changes and stressful events in a healthy way and it results in positive and beneficial outcomes after going through stressful event. In an agency, challenges are always present and it is the team leader's duty and responsibility to lead the whole team to overcome the difficulties. These challenges may become a driving force to team growth if the challenges are finally settled. As being stressful may be a good time for oneself or the whole group to grow, I took action to do something during the class. I am relatively weak in English, especially for oral English. I feel stressful when speaking English. However, as realizing my limitation and being encouraged by the teacher, I decide to take the initiative to express my opinion in English in class. This course gave me a chance to train up my attitude towards challenges and I think it would be very useful in my future career.

Here comes my personal feeling about the subject. It is good for the subject to use a lot of activities to facilitate our learning. This is a unique element of the subject as it uses an experiential learning approach. We could learn the qualities of a leader through participating in the activities. As a social work student, I am very familiar with such practice and joined the activities happily with my fellow classmates. The study mode is not familiar to those students from other departments but I thought they were interested in this study mode as it would make the learning more interesting and relaxing.

Another feeling is that there may be some room for course improvement. Before choosing this subject, I thought the course could include some outdoor activities such as adventure based training for us to train up our leadership. However, although the theoretical bases of the qualities of a leader were taught during the course, there was a lack of real practice time. The small games for classmates were not practical enough for us to better know such qualities. Although the worksheets provided could help us reflect our qualities of leadership, they did not give us enough practical experiences to reflect sometimes.

It is only the first trial of this subject in the university and it is inevitable for the course to have such minor limitations. I believe with improvement, the course could help more students know how to be a tomorrow's leader!

**Reflective Journal 3 (Lai Cho Ching)**

Each year, there are many graduates from the universities. Do you ever think of how to become an outstanding one? In order to prepare well, we need to raise our competitiveness as contemporary society sets an increasingly high standard for talents. Various qualities, such as analytic skills, communication skills and leadership, become more essential and significant. Among these qualities, leadership is an indispensable factor.

Leadership is about the communication among team members. It helps to achieve successful businesses and championship teams since it is believed that team work can bring a synergy effect to both work efficiency and productivity. Everyone wants to be a leader. How to be a good leader? Here is a chance for us to learn to lead people.

This year, an innovative General Education program, which is called “Tomorrow's Leaders,” was launched. “Tomorrow's leaders” gave me a precious opportunity to reinforce my personal development, especially for the leadership aspect. The subject really inspired me a lot not only in terms of theories and knowledge, but it also provided a valuable learning experience to me. It gave a comprehensive inspiration to my holistic personal development.

“Tomorrow's Leaders” was a relatively “unique” General Education subject. Instead of holding lectures in a boring and dull manner—sitting in the lecture hall and simply listening to what the lecturer says, “Tomorrow's Leaders” gave us interesting and unforgettable learning experience. Well-organised lectures were given to enable our understanding of various useful leadership theories. The leadership theories of basic personal qualities of effective leaders were integrated with different in-class activities. Various in-class activities were held to generate practical experiences. With in-class interactions, the frame of classroom disappeared. Besides learning from the professor, learning from our peers also benefited us a lot. Thus, we could learn in an interactive way. Not just for fun, the activities could effectively reflect our intrapersonal and interpersonal qualities. They gave us a better understanding of the theories. With those practical exercises, our self-awareness was developed. We could understand ourselves better by interacting with each other. Peers could point out the weaknesses of each other. Realizing our weaknesses, our specific leadership qualities were reinforced and improved during the fruitful in-class activities. Our learning efficiency was therefore greatly enhanced in the course.

“Tomorrow's Leaders” was also useful to our daily lives. We could apply leadership qualities that we learnt during the course in different aspects. Undoubtedly, the knowledge helped us to become a leader during team work. It also taught us how to be a leader in our own life—coping with life stresses instead of overwhelmed by them. University students faced a lot of pressure from study, part-time job, and so forth. The course definitely helped us to have better management in our lives. Emotional intelligence referred to the recognition of our own emotional state and the emotional states of others. It helped us to keep an optimistic attitude towards life. Negative situations need not be accompanied by negative emotions if we have a healthy perception. So, we can maintain our mental health and our lives will become easier. This quality is especially important nowadays. Under high work pressure, we have to control impulsive feelings and behaviors so that we can manage our emotions healthily. Time management was another important topic. It helped us control and allocate the amount of time spent on specific activities wisely. We have to do lots of things every day. So, we need to learn how to manage the task but not to be managed by the task. Not just doing things cautiously, it was also important to do things in an efficient and productive way.

In addition to in-class continuous assessment, group project gave us a good chance to practice what we have learned. Based on the topics we discussed during the lessons, each group needed to investigate the topics more deeply. It strengthened our knowledge in different leadership qualities. At the same time, our interpersonal skills were polished during the discussion. It gave us chances to practice the theories in our daily life. Since there were many members in one group, we learned to listen and accept different opinions. Members with different types of thinking could train us how to tolerate different opinions. It was certainly an important quality to become a wise leader—listening to different opinions. On the other hand, presentation was an effective learning process. Presenters could practice public speaking and have self-reflection, while audience could learn from others' good work. To have further improvement, professors gave comments based on our performance regarding the content and presentation skills. Thus, students could learn from mistakes and avoid them.

Ethical issue was another topic covered in “Tomorrow's Leaders.” This topic was an important topic but it was frequently ignored by people. Ethics determined the proper way of action for an individual. It was the framework for us to categorize our values and pursue them. It was also a controversial topic as different people might have different ethical standards. However, ethics played an important role in establishing reliability and reputation of ourselves. When we considered ethics when making decisions, we could build up our integrity and honesty. These were the best ways to let others trust and follow a leader. During the lessons, in-class activities about ethical struggling were launched. The “real” examples gave us a chance to understand our bottom line—what will we do if we really face this situation in the future? Peers may act differently under the same situation. Students could discuss and exchange their opinions. Although we might hold opposite opinions, it could act as a reminder in our future.

Undoubtedly, leadership is an irreplaceable quality in today's society. A good and responsible leader can promote communication among team members. He/she can help achieve successful businesses and championship teams since well-cooperated team work can bring a synergy effect to both work efficiency and productivity. “Tomorrow's Leaders” certainly gave me a precious opportunity to reinforce my different aspects of personal development, especially for the leadership aspect. Besides theories and knowledge, the valuable learning experience also inspired me a lot. It gave students a platform to explore their full potential and become a true leader.

**Reflective Journal 4 (Yuen Chi Kin)**

This is Calvin, a recipient of the Wofoo Foundation Scholarship 2010/2011 in the subject of “Tomorrow's Leaders,” which was offered by the Department of Applied Social Sciences. I am also a Year 2 student of the BSc (Hons) in Hotel Management program. I graduated from the Hong Kong Institute of Vocational Education with the Higher Diploma in International Hospitality Management. With outstanding results, I applied for the Bachelor programme through the Non-JUPAS system and I am now a Year 2 student at the School of Hotel and Tourism Management of The Hong Kong Polytechnic University. “Tomorrow's Leaders” was a General Education subject which attempted to broaden students' knowledge in other disciplines. This subject was brand new in the past semester, but I was attracted by the dynamic syllabus and the interactive teaching styles.

As a student who majored in hotel management, the study of leadership became particularly important. This subject covers most of the important topics that are required for a successful leader, or a manager. It covered knowledge from self-understanding to interpersonal communication and from ethics and morality to team building, with many topics that factually strengthen my knowledge to be a good and effective leader.

This is my second time to study a subject offered by the Department of Applied Social Sciences in this year. The first one was about psychology, and my second time here was also wonderful. You may have an idea that learning leadership is boring. However, the creative and interactive learning approaches broke this preconception. I can say this is the best subject that I have ever taken. The teaching team used different media and designed different games for every single topic, and student interaction and participation got at least one-third of the lecture time. It was not only about the knowledge from textbook. The lecturer always shared with us on the practical side, and we did have time to practice for each topic in a relaxing environment.

No matter for my study or my personal development, the subject is definitely beneficial to me. Getting familiar to myself serves as the basis of getting well with others, which further helps every student's growth in leadership in a great deal. For me, completion of this subject gave me a fresh and altered mind on my sense of being, everyday life and my future career plan. For example, my system of thinking has been changed, my approaches for processing data has been changed, I know how to control my emotions when I am under pressure and I got better approach to please others and form effective team work. All these will be valuable for correcting my personal mind, rewarding my study, strengthening my leadership skills, and probably shaping my future life.

The subject also required students to complete a group presentation and an individual assignment. It brought us a chance to interact with others and motivated us to seek in-depth knowledge in a particular topic through self-learning. For instance, my chosen topic was “Ethics and Morality,” which required us to associate it with the managerial and leadership styles. Diverse theories, models, cases studies and discussions were included in the presentation. We also presented recommendations on the organizational, personal and educational levels. By preparing the presentation and the individual assignment, we could all possess in-depth knowledge of this subject, and the presentation did provide valuable information to our classmates. At the same time, we also gained from the presentations conducted by other students, which included the topics of self-understanding, emotional competence, cognitive competence, resilience, spirituality, social competence, positive personal identity and interpersonal communication. These topics are required for being a successful and great leader. The teaching approach gave us a perfect and relaxing atmosphere to absorb the knowledge that we need. I would definitely recommend this subject to everyone that I know.

All in all, I am really glad that I had selected this subject during the subject registration and successfully completed it. The gladness is not only about getting a good grade of A+ but also receiving a scholarship that reduces my financial burden on the study. The truth is that I did enjoy studying this subject and I did enjoy it in every single lesson. I paid attention to every topic and kept thinking about that after the lecture. I strongly believe that it is beneficial to my personal development and my future career.

On the other hand, the scholarship serves as an acknowledgment of my hard work throughout the semester. It also becomes the driving force for my internship. By receiving the scholarship, the financial burden of my family was also relieved, and I can simply concentrate on my study and work for the best. The scholarship also supports me financially for my personal development. I have just completed a three-month overseas internship in this summer at the Walt Disney World, Orlando, Florida. I am also going to represent the School to attend a four-day industrial convention in Haikou and Sanya in late September.

Frankly speaking, the subject and the scholarship reinforced my faith to be a good leader in the future. It is not only about the one in the workplace, but also a good leader in the society. Lastly, I would like to take this opportunity to express my specific thanks to Wofoo Foundation for donating the scholarship that recognizes my hard work and motivates me to further excel in my academic performance. I would also like to thank the School of Hotel and Tourism Management and the Department of Applied Social Sciences of the Hong Kong Polytechnic University for offering me such an amazing and professional subject. Finally, I must thank Dr. Lit [my lecturer] and the teaching team for providing me the incredible knowledge and enjoyable learning atmosphere, and nominating me for the scholarship.

**Reflective Journal 5 (Tong Chun Wai)**

After three months, I still remembered what I have learnt in “Tomorrow's Leaders.” This module is really good for me because I did reflect my life experience and what I have learnt in this module. Also, I was very lucky that my teammates and I built up a good relationship during the class. They helped me find out my strengths and weaknesses. I am really thankful of this. This article is a good chance for me to share my feelings towards this subject. In the following parts, I will mainly share my feeling about the class schedule and how this subject has inspired me. At the same time, I will share my life experience and share about what I will do in order to be tomorrow's leader.

After I attended this subject, I found that this subject was not just a leadership training course but also a personal development training course for the young people. When I looked at the class schedule, many topics were about personal development such as self-understanding, emotional competence, resilience, social competence, team building and interpersonal communication, and so forth. These topics were about adolescent personal development and mainly focused on improving the weaknesses of contemporary young people.

When we read the newspapers or magazines today, we can find some articles criticizing the behavior of the post 80s, such as bad communication skills, bad emotional intelligence, weak social competence and weak adversity quotient. In fact, I am one of the post 80s and I cannot deny that these are not lies because I know that there are some common weaknesses of our generation. The reasons for our weaknesses may be due to the fact that we are too lucky to be born in a wealthy society and always protected by our families. For me, I am more concerned about how to improve these weaknesses instead of analyzing why I had these weaknesses. Therefore, I appreciate that the content of this course is trying to improve our weaknesses. In other words, this subject is well designed for the youth nowadays.

For university education, I agree the education should mainly focus on our professional skills training and help us prepare for our future careers. However, when we get into the university, most of us are adults already. So, it may be one of the best moments for us to think of our lives and ourselves. For example, we may need to think about our life goals. What can we do for the society? How do other people think of us? I really think that as a university student, we really need to think of these questions because we are not kids anymore and we will enter the work force very soon.

This subject I took really inspires me to critically think of these questions and prepares myself to enter the work force. One of the lessons I liked most was the first lesson which was about self-understanding. People may think that self-understanding is not for improving the weaknesses of young people or enhancing youth leadership skills. However, it does not make sense if we can enhance our leadership skills and improve our weaknesses but at the same time we do not understand ourselves. Personally, I think the fastest way to find out our weaknesses and our talents is trying to understand ourselves first. Many people think that they really understand themselves but it is not true because some of their weaknesses and their talents may be hard to find out by themselves. They need their friends, their families and their teachers to find out for them. After knowing our personality, it is easier to find out our weaknesses and talents. So, I consider self-understanding as the first step of being a successful leader.

Personally, this subject is very useful for me and inspires me in my life experience. I know that being a successful leader or improving my weaknesses is a life-long process but I will try my best to do this. The easiest way and most efficient way for me to step forward to become a leader is to enhance my communication skills. The reason behind is that good communication may be the only way for me and my future teammates to get familiar with each other. It is the only way for me to know how my teammates feel about me and the only way for me to know what I have done may not be good enough. I cannot imagine if there is no communication between the leader and team members but the team can still be effective and efficient.

For my whole life, especially being a leader, I would try to communicate with people around me like my family, my friends and my teammates in order to know about their feelings and opinions. I remembered when I was studying in Secondary 5, I was a school basketball team member. My teammates and I lost the first game in a match and everyone was quite depressed about it because we were the champion one year ago. We really doubted our ability to win the championship again in that year. However, my teammate and I decided to have a meeting after that match. We spent nearly 2 hours to discuss why we lost that game and we shared our opinions on team or teammates one by one. We figured out that we lacked communication in that match. Many mistakes caused the failure and some teammates even blamed others in that match. After listening to all views of the teammates, we set up some suggestions in order to win the next match. Finally, we beat the same team which won us in the first game in the final round, and we won the championship again. I really think that the meeting was a turning point because after that meeting, we knew that we still wanted to be the champion again and we improved ourselves.

Therefore, up till now, I have been trying to enhance my communication skills. I use different methods to communicate and I find that communication skills might not work every time but it works very often.

As a result, in my near future, I will try my best to find out some communication skills which are good for me to communicate with my teammates and others. I know that just communication skills are not enough for being a successful leader. Therefore, I would like to open my eyes to learn how to be a good and successful leader so that I can use those skills in my future careers and in my whole life.

## 2. Discussion

Four major observations could be highlighted. First, the students appreciated the course, including its content and design. Second, the students had very favourable perceptions of the instructors, including their knowledge, attitudes, and skills. Third, they perceived that the course was able to promote their holistic development—the course promoted their self-understanding as well as reflections and provided many opportunities for practice of skills. Finally, there are suggestions for refinement in the course which are also revealed by other evaluation strategies such as the post-lecture subjective outcome method. Of course, we should bear in mind the present findings are based on the Wofoo Foundation Scholarship recipients which may show the positive aspects of the program. While this possibility may exist, it is noteworthy that the present findings are generally in line with the evaluation findings based on objective outcome evaluation, subjective outcome evaluation, process evaluation, and qualitative evaluation strategies that the course was well received by the students, and it promoted the holistic development of students. Consistent with the evaluation findings based on Project P.A.T.H.S. based on junior secondary school students [6–12], the present study suggests that positive youth-development approach is a promising strategy to promote psychosocial competencies in university students.
